# Retraction Note: Case report on successful treatment for brain abscess in a Japanese monkey (*Macaca fuscata*)

**DOI:** 10.1186/s42826-026-00266-w

**Published:** 2026-01-29

**Authors:** Tohru Kimura

**Affiliations:** Laboratory Animal Science, Joint Faculty of Veterinary Medicine, Yamaguchi University, 1677-1, Yoshida, Yamaguchi, 753-8515 Japan

**Laboratory Animal Research (2023) 39:13**

10.1186/s42826-023-00165-4

The Editor-in-Chief has retracted this article. Originally concerns were raised about the presence of the animal ethics approval number in a case report and the claim that the experiment had been conducted at the National Institutes of Natural Sciences, not Yamaguchi University. The author was asked to provide an explanation and the ethics approval but did not reply. The author did not reply to correspondence from the Editor about this retraction.

